# 1-Nitro-4-(4-nitro­phen­oxy)benzene: a second monoclinic polymorph

**DOI:** 10.1107/S1600536813029346

**Published:** 2013-11-06

**Authors:** Mehwish Naz, Zareen Akhter, Vickie McKee, Arif Nadeem

**Affiliations:** a Department of Chemistry, Quaid-i-Azam University, Islamabad, Pakistan; b Chemistry Department, Loughborough University, Loughborough, LE11 3TU, England

## Abstract

In the title compound, C_12_H_8_N_2_O_5_, the aromatic rings are inclined to one another by 56.14 (7)°. The nitro groups are inclined by to the benzene rings to which they are attached by 3.86 (17) and 9.65 (15)°. In the crystal, mol­ecules are linked by C—H⋯O hydrogen bonds, forming a three-dimensional structure. The title compound is a new monoclinic polymorph, crystallizing in space group *P*2_1_/*c*. The first polymorph crystallized in space group *C*2/*c* and the mol­ecule possesses twofold rotation symmetry. Two low-temperature structures of this polymorph (150 K and 100 K, respectively) have been reported [Meciarova *et al.* (2004). Private Communication (refcode IXOGAD). CCDC, Cambridge, England, and Dey & Desiraju (2005). *Chem. Commun.* pp. 2486–2488].

## Related literature

For the crystal structure of the monoclinic *C*2/*c* polymorph of the title compound, see: Meciarova *et al.* (2004 ▶); Dey & Desiraju (2005 ▶).
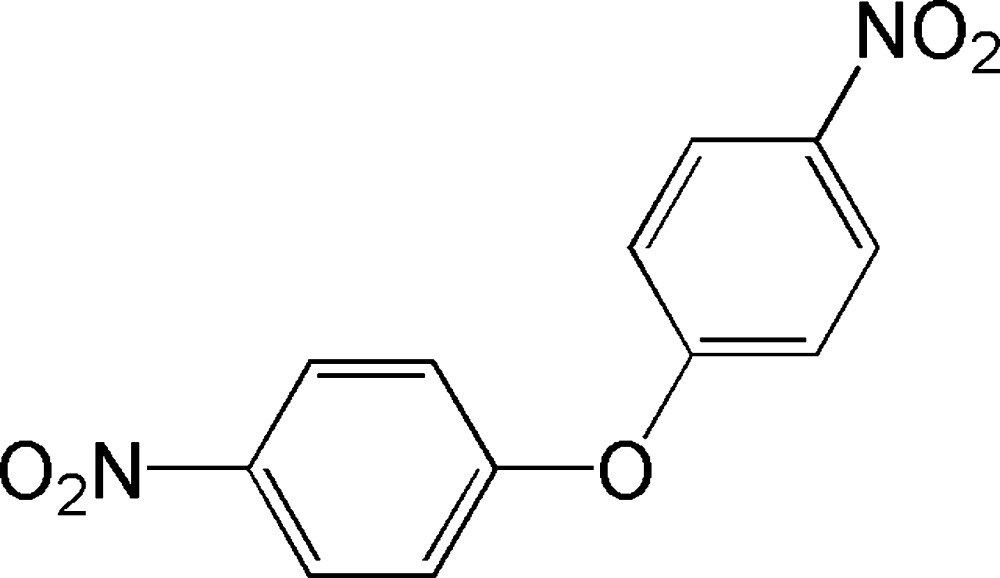


## Experimental

### 

#### Crystal data

C_12_H_8_N_2_O_5_
*M* *_r_* = 260.20
Monoclinic, 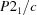

*a* = 8.1114 (5) Å
*b* = 11.8942 (7) Å
*c* = 12.3970 (7) Å
β = 106.402 (1)°
*V* = 1147.37 (12) Å^3^
*Z* = 4
Mo *K*α radiation
μ = 0.12 mm^−1^
*T* = 150 K
0.36 × 0.35 × 0.25 mm

#### Data collection

Bruker APEXII CCD diffractometer
Absorption correction: multi-scan (*SADABS*; Sheldrick, 2012 ▶) *T*_min_ = 0.794, *T*_max_ = 0.862
10808 measured reflections
2641 independent reflections
2149 reflections with *I* > 2σ(*I*)
*R*_int_ = 0.026

#### Refinement

*R*[*F* ^2^ > 2σ(*F* ^2^)] = 0.039
*wR*(*F* ^2^) = 0.104
*S* = 1.06
2641 reflections
172 parameters
H-atom parameters constrained
Δρ_max_ = 0.23 e Å^−3^
Δρ_min_ = −0.26 e Å^−3^

### 

Data collection: *APEX2* (Bruker 1998 ▶); cell refinement: *SAINT* (Bruker 1998 ▶); data reduction: *SAINT*; program(s) used to solve structure: *SHELXS97* (Sheldrick, 2008 ▶); program(s) used to refine structure: *SHELXL2012* (Sheldrick, 2008 ▶); molecular graphics: *SHELXTL* (Sheldrick, 2008 ▶) and *PLATON* (Spek, 2009 ▶); software used to prepare material for publication: *SHELXTL*.

## Supplementary Material

Crystal structure: contains datablock(s) I. DOI: 10.1107/S1600536813029346/ds2235sup1.cif

Structure factors: contains datablock(s) I. DOI: 10.1107/S1600536813029346/ds2235Isup2.hkl

Supplementary material file. DOI: 10.1107/S1600536813029346/ds2235Isup3.cml

968365

Additional supplementary materials:  crystallographic information; 3D view; checkCIF report

## Figures and Tables

**Table 1:** Hydrogen-bond geometry (Å, °)

*D*—H⋯*A*	*D*—H	H⋯*A*	*D*⋯*A*	*D*—H⋯*A*
C13—H13⋯O21^i^	0.95	2.56	3.4413 (18)	153
C23—H23⋯O11^ii^	0.95	2.31	3.1933 (18)	154
C26—H26⋯O12^iii^	0.95	2.42	3.227 (2)	143

Symmetry codes: (i) 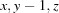; (ii) 
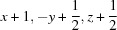
; (iii) 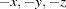.

**Fig. 1.**
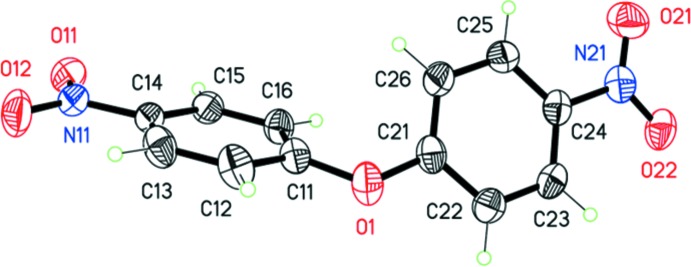
A view of the molecule structure of the title molecule. Displacement ellipsoids are drawn at the 50% probability level.

**Fig. 2.**
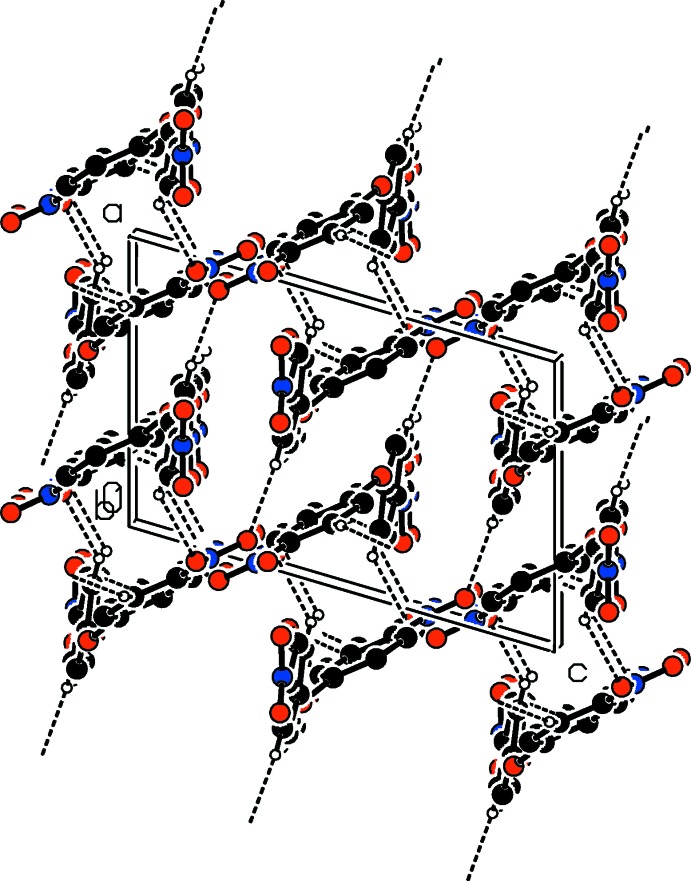
A view along the b axis of the crystal packing of the title compound. The C-H···O hydrogen bonds are shown as dashed lines (see Table 1 for details).

## supplementary crystallographic information

### 1. Comment

The molecular structure of the title molecule is illustrated in Fig. 1. The molecule has a a twisted V-shape with the two nitrophenyl rings being inclined to one another by 56.14 (7) °. The nitro group N11/O11/O12 is inclined to the benzene ring C11-C16, to which it is attached, by 3.86 (17) °, while the nitro group N21/O21/O22 is inclined to benzene ring C21-C26 by 9.65 (15) °.

In the crystal, molecules are linked by C-H···O hydrogen bonds forming a three-dimensional structure (Table 1 and Fig. 2).

The title compound is a new monoclinic polymorph, crystallizing in space group P2_1_/c. The first polymorph crystallized in space group C2/c and the the molecule possesses two-fold rotation symmetry with the ether O atom lying on the two-fold rotation axis. The 150 K structure was reported on by (Meciarova *et al.*, 2004), while the 100 K structure was reported on by (Dey & Desiraju, 2005). Taking the 100 K structure as reference it can be seen that the molecular structure is slightly different to that of the present polymorph with the two nitrophenyl rings being inclined to one another by 66.75 (6) ° and the nitro group being inclined to the benzene ring by 12.97 (13) °. In the crystal, molecules are also linked by C—H···O hydrogen bonds forming a three-dimensional structure.

### 2. Experimental

A mixture of 4-nitrochlorobenzene (38.2 mmol), 1,6-hexan-diol (19.1 mmol) and anhydrous potassium carbonate (38.2 mmol) in 50 ml of THF was placed in a three necked prebaked round bottom flask fitted with a reflux condenser, a nitrogen inlet and a magnetic stirrer. After refluxing for 18 h in an inert atmosphere, the mixture was cooled to room temperature and poured into 400 ml of water, yielding a yellow solid. The product was filtered, dried and recrystallized from ethanol. The title compound was obtained as a by-product in the form of block-like yellow crystals [yield 10%; M.p. = 416 K].

### 3. Refinement

C-bound H atoms were included in calculated positions and refined as riding atoms: C-H = 0.95 Å with U_iso_(H) = 1.2U_eq_(C).

### Crystal data

**Table:**

C_12_H_8_N_2_O_5_	*F*(000) = 536
*M**_r_* = 260.20	*D*_x_ = 1.506 Mg m^−^^3^
Monoclinic, *P*2_1_/*c*	Melting point: 416 K
Hall symbol: -P 2ybc	Mo *K*α radiation, λ = 0.71073 Å
*a* = 8.1114 (5) Å	Cell parameters from 3529 reflections
*b* = 11.8942 (7) Å	θ = 2.4–30.6°
*c* = 12.3970 (7) Å	µ = 0.12 mm^−^^1^
β = 106.402 (1)°	*T* = 150 K
*V* = 1147.37 (12) Å^3^	Block, yellow
*Z* = 4	0.36 × 0.35 × 0.25 mm

### Data collection

**Table:**

Bruker APEXII CCD diffractometer	2149 reflections with *I* > 2σ(*I*)
Radiation source: fine-focus sealed tube	*R*_int_ = 0.026
ω rotation with narrow frames scans	θ_max_ = 27.5°, θ_min_ = 2.4°
Absorption correction: multi-scan (*SADABS*; Sheldrick, 2012)	*h* = −10→10
*T*_min_ = 0.794, *T*_max_ = 0.862	*k* = −15→15
10808 measured reflections	*l* = −16→16
2641 independent reflections	

### Refinement

**Table:**

Refinement on *F*^2^	0 restraints
Least-squares matrix: full	Hydrogen site location: inferred from neighbouring sites
*R*[*F*^2^ > 2σ(*F*^2^)] = 0.039	H-atom parameters constrained
*wR*(*F*^2^) = 0.104	*w* = 1/[σ^2^(*F*_o_^2^) + (0.0456*P*)^2^ + 0.2844*P*] where *P* = (*F*_o_^2^ + 2*F*_c_^2^)/3
*S* = 1.06	(Δ/σ)_max_ < 0.001
2641 reflections	Δρ_max_ = 0.23 e Å^−^^3^
172 parameters	Δρ_min_ = −0.26 e Å^−^^3^

### Special details

**Table:**

Geometry. All e.s.d.'s (except the e.s.d. in the dihedral angle between two l.s. planes) are estimated using the full covariance matrix. The cell e.s.d.'s are taken into account individually in the estimation of e.s.d.'s in distances, angles and torsion angles; correlations between e.s.d.'s in cell parameters are only used when they are defined by crystal symmetry. An approximate (isotropic) treatment of cell e.s.d.'s is used for estimating e.s.d.'s involving l.s. planes.

### Fractional atomic coordinates and isotropic or equivalent isotropic displacement parameters (Å^2^)

**Table:**

	*x*	*y*	*z*	*U*_iso_*/*U*_eq_	
O1	0.44094 (13)	0.19652 (8)	0.09931 (8)	0.0438 (3)	
C11	0.32514 (17)	0.12641 (11)	0.02667 (11)	0.0346 (3)	
C12	0.3348 (2)	0.01415 (11)	0.05873 (12)	0.0427 (3)	
H12	0.4100	−0.0082	0.1290	0.051*	
C13	0.2350 (2)	−0.06452 (11)	−0.01166 (12)	0.0414 (3)	
H13	0.2406	−0.1416	0.0092	0.050*	
C14	0.12673 (16)	−0.02940 (10)	−0.11323 (11)	0.0341 (3)	
N11	0.02465 (15)	−0.11328 (10)	−0.18957 (11)	0.0427 (3)	
O11	−0.07448 (13)	−0.08066 (10)	−0.27901 (11)	0.0564 (3)	
O12	0.04440 (16)	−0.21295 (9)	−0.16171 (11)	0.0581 (3)	
C15	0.11403 (17)	0.08250 (11)	−0.14550 (12)	0.0361 (3)	
H15	0.0368	0.1048	−0.2151	0.043*	
C16	0.21520 (17)	0.16134 (10)	−0.07512 (11)	0.0349 (3)	
H16	0.2095	0.2384	−0.0961	0.042*	
C21	0.41096 (18)	0.31038 (11)	0.10392 (11)	0.0353 (3)	
C22	0.55556 (17)	0.37841 (12)	0.12879 (11)	0.0369 (3)	
H22	0.6661	0.3464	0.1384	0.044*	
C23	0.53798 (17)	0.49294 (11)	0.13949 (10)	0.0359 (3)	
H23	0.6357	0.5408	0.1552	0.043*	
C24	0.37637 (17)	0.53685 (11)	0.12704 (10)	0.0329 (3)	
N21	0.35664 (16)	0.65877 (9)	0.13676 (9)	0.0388 (3)	
O21	0.21649 (15)	0.69525 (9)	0.13919 (11)	0.0592 (3)	
O22	0.48044 (14)	0.71924 (8)	0.14138 (9)	0.0477 (3)	
C25	0.23130 (18)	0.46974 (12)	0.10442 (12)	0.0394 (3)	
H25	0.1215	0.5018	0.0975	0.047*	
C26	0.24867 (18)	0.35511 (12)	0.09212 (13)	0.0420 (3)	
H26	0.1507	0.3075	0.0758	0.050*	

### Atomic displacement parameters (Å^2^)

**Table:**

	*U*^11^	*U*^22^	*U*^33^	*U*^12^	*U*^13^	*U*^23^
O1	0.0519 (6)	0.0328 (5)	0.0393 (5)	0.0013 (4)	0.0010 (4)	−0.0048 (4)
C11	0.0417 (7)	0.0300 (6)	0.0332 (6)	−0.0018 (5)	0.0122 (5)	−0.0024 (5)
C12	0.0603 (9)	0.0338 (7)	0.0329 (7)	0.0052 (6)	0.0115 (6)	0.0050 (5)
C13	0.0597 (9)	0.0258 (6)	0.0430 (8)	0.0006 (6)	0.0217 (7)	0.0047 (5)
C14	0.0336 (6)	0.0294 (6)	0.0435 (7)	−0.0040 (5)	0.0176 (6)	−0.0047 (5)
N11	0.0383 (6)	0.0374 (6)	0.0580 (8)	−0.0086 (5)	0.0227 (6)	−0.0107 (6)
O11	0.0338 (5)	0.0551 (7)	0.0719 (8)	−0.0026 (5)	0.0012 (5)	−0.0181 (6)
O12	0.0775 (8)	0.0332 (6)	0.0725 (8)	−0.0187 (5)	0.0357 (7)	−0.0101 (5)
C15	0.0343 (7)	0.0336 (7)	0.0393 (7)	0.0009 (5)	0.0084 (5)	0.0012 (5)
C16	0.0406 (7)	0.0254 (6)	0.0381 (7)	−0.0007 (5)	0.0102 (5)	0.0035 (5)
C21	0.0466 (7)	0.0309 (6)	0.0280 (6)	−0.0022 (5)	0.0098 (5)	−0.0016 (5)
C22	0.0358 (7)	0.0425 (7)	0.0290 (6)	0.0004 (6)	0.0035 (5)	−0.0008 (5)
C23	0.0371 (7)	0.0388 (7)	0.0294 (6)	−0.0090 (5)	0.0055 (5)	−0.0011 (5)
C24	0.0433 (7)	0.0308 (6)	0.0253 (6)	−0.0057 (5)	0.0106 (5)	0.0002 (5)
N21	0.0512 (7)	0.0340 (6)	0.0327 (6)	−0.0057 (5)	0.0142 (5)	0.0016 (5)
O21	0.0606 (7)	0.0381 (6)	0.0882 (9)	0.0030 (5)	0.0362 (7)	0.0020 (6)
O22	0.0576 (6)	0.0370 (5)	0.0488 (6)	−0.0158 (5)	0.0153 (5)	−0.0031 (4)
C25	0.0384 (7)	0.0375 (7)	0.0458 (8)	−0.0033 (6)	0.0174 (6)	−0.0044 (6)
C26	0.0420 (7)	0.0370 (7)	0.0511 (8)	−0.0107 (6)	0.0196 (6)	−0.0085 (6)

### Geometric parameters (Å, º)

**Table:**

O1—C21	1.3799 (16)	C16—H16	0.9500
O1—C11	1.3824 (16)	C21—C22	1.3861 (19)
C11—C16	1.3873 (18)	C21—C26	1.389 (2)
C11—C12	1.3890 (19)	C22—C23	1.3801 (19)
C12—C13	1.376 (2)	C22—H22	0.9500
C12—H12	0.9500	C23—C24	1.3787 (19)
C13—C14	1.381 (2)	C23—H23	0.9500
C13—H13	0.9500	C24—C25	1.3835 (18)
C14—C15	1.3852 (18)	C24—N21	1.4677 (17)
C14—N11	1.4608 (17)	N21—O22	1.2233 (15)
N11—O12	1.2325 (16)	N21—O21	1.2251 (16)
N11—O11	1.2340 (17)	C25—C26	1.384 (2)
C15—C16	1.3819 (18)	C25—H25	0.9500
C15—H15	0.9500	C26—H26	0.9500
			
C21—O1—C11	121.58 (10)	O1—C21—C22	115.73 (12)
O1—C11—C16	123.77 (12)	O1—C21—C26	122.99 (12)
O1—C11—C12	114.87 (12)	C22—C21—C26	121.14 (12)
C16—C11—C12	121.19 (12)	C23—C22—C21	119.58 (13)
C13—C12—C11	119.79 (13)	C23—C22—H22	120.2
C13—C12—H12	120.1	C21—C22—H22	120.2
C11—C12—H12	120.1	C24—C23—C22	118.95 (12)
C12—C13—C14	118.77 (12)	C24—C23—H23	120.5
C12—C13—H13	120.6	C22—C23—H23	120.5
C14—C13—H13	120.6	C23—C24—C25	122.11 (12)
C13—C14—C15	122.02 (12)	C23—C24—N21	119.17 (11)
C13—C14—N11	118.84 (12)	C25—C24—N21	118.72 (12)
C15—C14—N11	119.13 (12)	O22—N21—O21	123.08 (12)
O12—N11—O11	123.66 (13)	O22—N21—C24	118.53 (12)
O12—N11—C14	118.06 (13)	O21—N21—C24	118.38 (11)
O11—N11—C14	118.27 (12)	C24—C25—C26	118.92 (13)
C16—C15—C14	119.16 (12)	C24—C25—H25	120.5
C16—C15—H15	120.4	C26—C25—H25	120.5
C14—C15—H15	120.4	C25—C26—C21	119.27 (12)
C15—C16—C11	119.06 (12)	C25—C26—H26	120.4
C15—C16—H16	120.5	C21—C26—H26	120.4
C11—C16—H16	120.5		

### Hydrogen-bond geometry (Å, º)

**Table:**

*D*—H···*A*	*D*—H	H···*A*	*D*···*A*	*D*—H···*A*
C13—H13···O21^i^	0.95	2.56	3.4413 (18)	153
C23—H23···O11^ii^	0.95	2.31	3.1933 (18)	154
C26—H26···O12^iii^	0.95	2.42	3.227 (2)	143

Symmetry codes: (i) *x*, *y*−1, *z*; (ii) *x*+1, −*y*+1/2, *z*+1/2; (iii) −*x*, −*y*, −*z*.
